# Variation of the encoding hyaluronic receptors Hyaluronan-mediated motility receptor (rs299295) and Stabilin-2 (rs2271637) genes with prostate neoplasms risk: A case-control and in silico study

**DOI:** 10.18502/ijrm.v23i3.18776

**Published:** 2025-06-10

**Authors:** Hayder Abdulhadi Saleh Albdairi, Abasalt Hosseinzadeh Colagar

**Affiliations:** Department of Molecular and Cell Biology, Faculty of Basic Science, University of Mazandaran, Babolsar, Iran.

**Keywords:** FAM83D, Fasciclin-like, HMMR, MAPK1, Prostate neoplasm, STAB2.

## Abstract

**Background:**

Hyaluronan-mediated motility receptor (HMMR) and Stabilin-2 (STAB2), known as extracellular matrix cell surface protein's receptors, bind to hyaluronic acid and lead to various cell functions.

**Objective:**

The study aims to investigate the relationship between the *HMMR*-rs299295 (C > T/ A485V) and *STAB2*-rs2271637 (C > G/ L2401V) gene variants and the risk of prostate neoplasms in the Mazandaran population, North of Iran.

**Materials and Methods:**

This study was conducted based on a case-control and *in silico* approach. Genomic DNA was extracted from 598 intravenous blood samples, collected from 250 benign prostatic hyperplasia (case group I) and 250 malignant prostate (case group II) neoplasms as cases, and 98 healthy men as control. The *HMMR*-rs299295 and *STAB2*-rs2271637 genotypes were identified using the polymerase chain reaction-restriction fragment length polymorphism method. Bioinformatics analyses were conducted using PolyPhen-2, GOR IV, and GeneMANIA free web tools.

**Results:**

The study found that the mutant T allele in *HMMR*-rs299295 and the G allele in *STAB2*-rs2271637 are associated with an increased risk of prostate neoplasm, including benign prostatic hyperplasia and prostate cancer (p < 0.001). Bioinformatic analyses revealed structural changes and potential damage from these variants. The *HMMR*-A485V variant might impair interaction with family with sequence similarity 83 member D, and the *STAB2*-L2401V variant could disrupt domain 7 of FAS1, together they may affect the protein's physical interactions, especially with mitogen-activated protein kinase 1.

**Conclusion:**

The mutant alleles of T in *HMMR*-rs299295 and the G in *STAB2*-rs2271637 may disrupt protein structures and probably contribute to prostate neoplasm progression.

## 1. Introduction

Prostate neoplasms are a term for abnormal growths in the prostate gland, a walnut-sized gland in men responsible for producing seminal fluid. Prostate neoplasms include benign prostatic hyperplasia (BPH) and prostate cancer (1). BPH is a common condition in older men that involves the non-cancerous enlargement of the prostate gland. Prostate cancer multiplies uncontrollably and forms malignant tumors, which are the most prominent and concerning compared to BPH. Prostate cancer is the second most frequently diagnosed cancer in men (2).

The advancement of prostate cancer is influenced by specific compounds found in the extracellular matrix (ECM), which is a complex structure composed of proteins and polysaccharides that encase cells. ECM components (such as collagen, fibronectin, laminin [3], and glycosaminoglycans [4]) play a crucial role in tumor cell behavior (5). Hyaluronic acid (HA) is a major component of the ECM and plays a critical role in maintaining tissue hydration, elasticity, and structural integrity. It is composed of repeating disaccharide units of D-glucuronic acid and N-acetyl-D-glucosamine that play a multifaceted role. It contributes to the tumor microenvironment by modulating cell proliferation, migration, and invasion (6). HA interacts with cell surface receptors, including cluster of differentiation 44, lymphatic vessel endothelial hyaluronan receptor 1, layilin, Stabilin-2 (STAB2), and hyaluronan-mediated motility receptor (HMMR) (7).

The *HMMR* gene, also known as the receptor for hyaluronan-mediated motility gene, is located on chromosome 5q32. It spans approximately 67 kilobases (kb) of genomic DNA and contains 18 exons (8). The *HMMR* gene is transcribed into mRNA with a length of around 5.2 kb. The coding sequence of this mRNA, which translates into the HMMR protein, is approximately 2.7 kb long. The mRNA undergoes several post-transcriptional modifications, including splicing, polyadenylation, and capping, which are essential for its stability and translation efficiency (8, 9). The HMMR protein, consisting of 725 amino acids and weighing about 84 kDa (8), has multiple domains. HMMR is essential for cell movement and division, aiding in cell motility and proliferation during tissue repair and remodeling by activating signaling pathways like mitogen-activated protein kinase (MAPK) and PI3K/Akt (7, 10).

The *STAB2* gene, also known as STAB2, is located on chromosome 12q13.3. It spans approximately 400 kb of genomic DNA and contains 65 exons. The mRNA transcribed from the *STAB*2 gene is about 9.5 kb long. The coding sequence of this mRNA, which translates into the STAB2 protein, spans approximately 7.5 kb (11). The STAB2 protein, a 300 kDa glycoprotein with 2552 amino acids, includes fasciclin, epidermal growth factor-like, and X-link domains for ligand interactions, and a transmembrane domain for receptor functions in endocytosis of HA (12). Functionally, STAB2 is essential for the scavenging and clearance of HA and other glycosaminoglycans from the ECM (12, 13). This process is vital for maintaining tissue homeostasis and preventing the accumulation of these molecules, which could lead to pathological conditions (14). Interaction of the HMMR and STAB2 proteins with the HA of ECM led to the promotion of cell motility and proliferation, processes that are vital during tissue repair and remodeling. Conversely, mutations in one of these proteins could be associated with increased cell neoplasms. One of the important mutations in the human population is single-nucleotide polymorphism (SNP) (15).

Thus, gene variations might specifically influence the structure of HMMR and STAB2 receptors. These ECM receptors are like docking stations that allow cells to communicate with the surrounding matrix such as HA. Since then, changes in receptor structures due to gene SNPs could disrupt this communication, potentially leading to an environment more conducive to prostate cancer progression. The study aims to investigate 2 gene variants, *HMMR*-rs299295 and *STAB2*-rs2271637, with the risk of prostate neoplasms based on a case-control and *in silico* study in the Mazandaran province (Iran) population.

## 2. Materials and Methods

This case-control study was conducted in Mazandaran province (Iran). A total of 598 samples were analyzed, including 545 archived blood samples collected between September 2017 and May 2023, which were donated by Moudi et al. (16), and 53 new samples collected during this study period (March to September 2024). Participants were recruited from 3 hospitals in Babol (Shahid Beheshti, Babol Clinic, and Rhohani hospital). Participants resided in Mazandaran province, mainly in the cities of Amol, Babol, and Qaem Shahr, Iran.

The study included 3 groups: one control and 2 case groups (including BPH or case group I and cancer or case group II). Participants in the control group were healthy individuals with no history of prostate issues, normal digital rectal examination (DRE) results (DRE score ≤ 3), and prostate-specific antigen (PSA) levels < 4 ng/ml. They were recruited during annual health checkups. The case group I consisted of men with PSA levels < 4 ng/ml, positive DRE (DRE score > 3), and a confirmed diagnosis following surgical intervention. Lastly, the case group II comprised men with PSA levels > 4 ng/ml, positive DRE, and Gleason scores ≥ 7. All participants were men aged > 50 yr with no prior history of prostate symptoms, aside from those specifically identified within the case group I and case group II. The exclusion criteria were individuals < 50 yr of age, those with prior prostate surgeries, or those with diagnosed chronic prostate conditions. Also, they included individuals with incomplete medical records or those who declined to participate. Data were extracted from the medical records of eligible participants.

### Sample size

To determine our sample size, we employed the following formula: 


$$n=\frac{{{\left(\frac{Z_{\alpha }}{2}+Z_{\beta }\right)}^{2}\times \left[P_{1}\left(1-P_{1}\right)+P_{2}\left(1-P_{2}\right)\right]}^{2}}{{\left(P_{1}-P_{2}\right)}^{2}}$$

According to this formula n = required sample size per group; Zα/2 = Z-score for the desired confidence level (1.96 for 95% confidence); Zβ = Z-score for desired power (0.84 for 80% power); P1 and P2 = expected proportions of the polymorphism in case groups and healthy controls, respectively. According to the NCBI data bank, the global frequencies of the C allele as wild type and the T allele as a mutant allele for *HMMR*-rs299295 are 0.764449 and 0.235551, respectively. For *STAB2*-rs2271637, the global frequencies of the C allele as wild type and the T allele as a mutant allele are 0.738826 and 0.261174, respectively. Also, this study considered both α error (at 0.05, indicating a 5% risk) and β error (0.20) in our study design. In our study, we did not conduct interim analyses or establish formal stopping guidelines. Given the nature of our investigation, which focused on genetic polymorphisms rather than treatment efficacy, we deemed interim analyses unnecessary. However, if future studies involve therapeutic interventions or more complex designs, we will consider implementing interim analyses to evaluate early efficacy or futility based on pre-defined criteria. Based on this approach, a total of 598 blood samples were collected and analyzed, comprising 250 samples from case group I, 250 samples from case group II, and 98 samples from the control group. Following venipuncture, peripheral blood samples (2 ml) were collected from all participants into laboratory tubes with ethylenediaminetetraacetic acid disodium salt dihydrate as an anticoagulant and stored at -20 C.

### DNA extraction and genotyping

**Figure 1:**
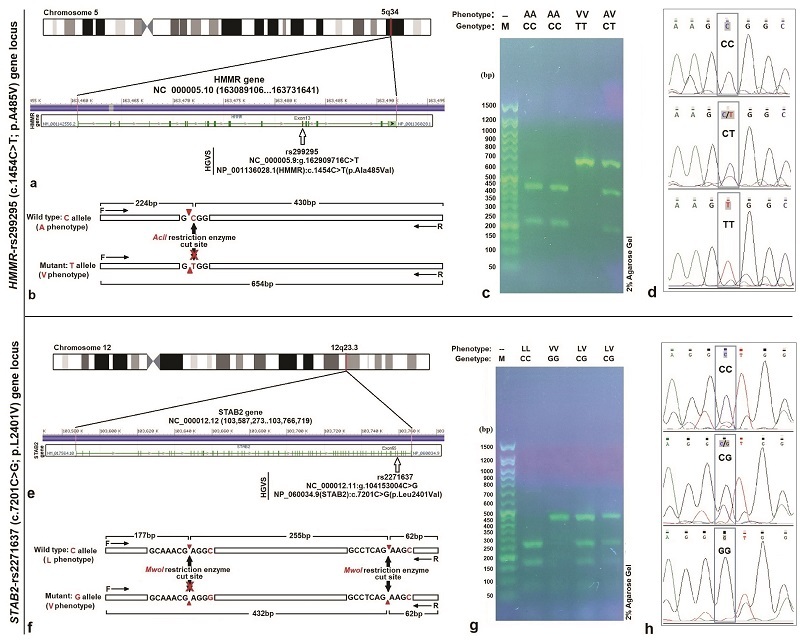
*HMMR*-rs299295 and *SATB2*-rs2271637 gene variants loci, RFLP maps, agarose gel profiling of PCR products digestion, and DNA sequencing results: a and e) The location of *HMMR* (chromosome 5) and *STAB2* (chromosome 12) genes which include exons 13 and 65 for *HMMR*-rs299295 and *SATB2*-rs2271637 variants, respectively, b and f) Schematic RFLP maps of *HMMR*-rs299295 and *STAB2*-rs2271637 showing restriction enzyme cleavage sites, c and g) Digestion with *AciI* and *MwoI* restriction enzymes revealed distinct band patterns on a 2% agarose gel. These patterns corresponded to the genotypes of *HMMR*-rs299295 (CC, CT, TT) and *STAB2*-rs2271637 (CC, CG, GG), respectively. M denotes 100 bp DNA marker (SinaColon Co., Iran), d and h) Sequencing chromatograms represented *HMMR*-rs299295 and *STAB2*-rs2271637 genotypes.

The extraction of DNA was performed with the SinaPure^TM^ DNA kit (EX6001) according to the provided protocol. Using polymerase chain reaction-restriction fragment length polymorphism (PCR-RFLP), the genotypes of *HMMR*-rs299295 and *STAB2*-rs2271637 were identified. Primer3Plus (https://www.ncbi.nlm.nih.gov/tools/primer-blast/) was employed to design PCR primers flanking the SNP, based on information from the NCBI database (Table I, Figure 1). Using an Eppendorf 5331 Gradient MasterCycler (Eppendorf Co., Germany), PCR was conducted with 2X, PCR MasterMix (SinaClon Co., Iran) in a 25 μL final volume, containing 10 pM of each primer and 20–100 ng DNA template (Table I).

For genotyping, amplified PCR products were digested with *AciI* restriction enzyme, which recognizes the sequence C ∧ CGC for the *HMMR*-rs299295 locus, and *MwoI* restriction enzyme, which recognizes the sequence GCNNNNN ∧ NNGC for *STAB2*-rs2271637 locus. Digested profiles of *AciI* generated 654, 430, and 224 bp fragments, and digested profiles of *MwoI* produced 432 bp, 255, 177, and 62 bp fragments, which these schematic RFLP maps showed in figure 1b and f. To analyze the size of PCR products and digested fragments, they were separated by electrophoresis on a 2% agarose gel. The gel was stained by immersing it in the 1 μg/mL ethidium bromide solution for visualization under a UV-transilluminator (ProteinSimple Red, Co., USA). 3 samples were randomly chosen from each case and control group to ensure genotype identification and submitted for sequencing (GenFanAvaran Co., Iran). Preparation of agarose gel, agarose gel electrophoresis in the Tris-Borat- ethylenediaminetetraacetic acid disodium salt dihydrate, staining of agarose gel, and digestion of PCR fragments performed by Green and Sambrook methods (17).

### Data sources for bioinformatic analysis

The functional impact and potential pathogenicity of *HMMR*-rs299295 and *STAB2*-rs2271637 were evaluated using PolyPhen-2 (http://genetics.bwh.harvard.edu/pph2), which predicts the effect of mutations on protein function. Additionally, GOR IV (https://npsa-prabi.ibcp.fr/) and Rosetta local distance difference test (LDDT) prediction (https://swissmodel.expasy.org/lddt) analyzed the potential secondary structural changes and assessed the reliability of protein structure models, particularly in terms of their local structural accuracy induced by the SNPs. GeneMANIA (http://genemania.org), a comprehensive web-based platform for predicting gene functions and interactions, was utilized in this research to determine the direct and co-direct physical associations between HMMR and STAB2 (18).

**Table 1:** Oligomers were used as primers and PCR conditions

**Genes**	**Primer names**	**Oligomers (5 '→ 3 ' )**	**Cycles and thermal conditions**	**PCR product**
*HMMR*	*F-rs299295*	5 ' GCCTCAGAACACTGAATTCTTAC	4 min at 94 C; 35 cycles (45 sec at 94 C, 30 sec at 59 C, 30 sec 72 C); finally, 10 min at 72 C	654 (bp)
	*R-rs299295*	5 ' TCTTCCTCCTGTTGCTTGAGTTG		
*STAB2*	*F-rs2271637*	5 ' GCAATTTTCTTCGTCATCCCCATC		654 (bp)
	*R-rs2271637*	5 ' CCAAAATGAAGGCGGAGAATCAG		
PCR: Polymerase chain reaction, *HMMR*: Hyaluronan-mediated motility receptor, *STAB2*: Stabilin-2, Oligomers: Sequence of primers, bp: Base pair (all primers were synthesized by CinnaClon Co., Iran)				

### Ethical Considerations

This study was approved by the Mazandaran University of Medical Science Ethics Committee, Mazandaran, Iran and conducted in accordance with the Iran National Committee for Ethics in Biomedical Researches (Code: IR.UMZ.REC.1403.011). This study was conducted at the University of Mazandaran, Babolsar, Iran. All subjects signed an informed consent form before entering the study. All personal data, such as names and contact details, were anonymized using unique identification codes. Access to the data was restricted to authorized members of the research team, and all information is securely stored. Additionally, no identifying information has been disclosed in the publication of the results.

### Statistical Analysis

In this study, Hardy-Weinberg equilibrium (HWE) was evaluated using a χ ² test. The online tool (https://wpcalc.com/en/equilibrium-hardy-weinberg/) was employed to compare the observed and expected genotype frequencies. Logistic regression analyses were used to compare the allele types and genotype distributions of *HMMR*-rs299295 and *STAB2*-rs2271637 between case groups (I and II) and healthy controls. The odds ratios and 95% confidence intervals were calculated. The Chi-square test and Fisher's exact test were also conducted to compare the control group with the case groups I and II. The p-value was calculated for the Chi-square test for genotypes with df = 2 and alleles with df = 1. In all statistical analyses, p < 0.05 was considered significant. All statistical analyses were performed by SPSS version 19 (PSS Inc., IBM Corp Armonk, NY, USA).

## 3. Results

### Genotype frequency

Digestion of amplified DNA fragments with the *AciI* enzyme and subsequent analysis on agarose gel revealed 3 genotypes and phenotypes for the *HMMR*-rs299295 variant. They displayed band patterns corresponding to TT (654 bp) with phenotype VV, CC (430 bp and 224 bp) with phenotype AA, and CT (654 bp, 430 bp, and 224 bp) with phenotype AV. DNA sequencing corroborated these findings (Figure 1c and d). Following *MwoI* enzyme digestion and agarose gel electrophoresis, genotyping of the *STAB2*-rs2271637 locus revealed 3 genotypes and phenotypes: GG (432 bp) with phenotype VV, CC (255, 177, and 62 bp) with phenotype LL, and CG (432, 255, 177, and 62 bp) with phenotype LV. DNA sequencing confirmed these genotypes (Figure 1g and h).

The results of the statistical analysis indicated a significant difference in the distribution of the CT, TT, and CT+TT genotypes of *HMMR*-rs299295 between the control group and both the case groups I and II, with p < 0.001 (Table II). Furthermore, a marked difference was observed in the frequency of the mutant allele T for *HMMR*-rs299295, which was found to be 12.4% in the control group compared to 52.4% in case group I and 44.4% in case group II, with p < 0.001 (Table II). Statistical analysis revealed significant differences in genotype frequencies (CG, GG, and CG+GG) of the *STAB2*-rs2271637 variant between the control and case group I (Table II).

P < 0.001, 0.022, and < 0.001, respectively. A significant difference was observed in the CG, GG, and CG+GG genotypes of *STAB2*-rs2271637 between the control and case groups II, with p = 0.005, 0.003, and < 0.001, respectively (Table II). Moreover, the frequency of the mutant allele G of *STAB2*-rs2271637 was significantly different between the control group (21.2%) and both the case group I (37.8%) and group II (31%), with p < 0.001 (Table II).

### Bioinformatics analysis

PolyPhen-2 was utilized to assess the effect of the amino acid substitution on protein function, with the results indicating potential damage. The scoring system ranges from 0–1.0, where higher values correspond to a higher probability of damage. For the *STAB2*-rs2271637 variant, PolyPhen-2 reported a leucine-to-valine substitution (L2401V) with a score of 0.898, which is predicted to be possibly damaging. Conversely, the *HMMR*-rs299295 variant leads to the substitution of alanine with valine at position 485 (A485V). This variant is predicted to be benign with a score of 0.0.

**Figure 2:**
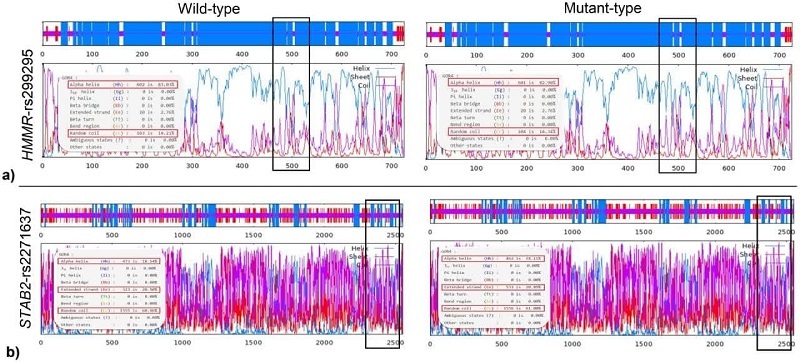
Predicted changes by GOR IV server in protein secondary structure for HMMR-rs299295 and STAB2-rs2271637 variants: a) GOR IV predicts slight alpha helix and random coil content changes for the HMMR-rs299295 variant (alanine vs. valine), b) GOR IV predicts the wild-type STAB2 protein (leucine) to have 18.5% alpha helix, 20.5% extended strand, and 61.0% random coil. The mutant (valine) shows a slight decrease in alpha helix and a small increase in the extended strand and random coil.

The GOR IV server, employed in our study, predicts the secondary structure of proteins based on their amino acid composition. Analysis using the *HMMR*-rs299295 variant server revealed changes in the protein's secondary structure. The wild-type (alanine) has 83.03% alpha helix and 14.21% random coil content, while the mutant (valine) has 82.90% alpha helix and 14.34% random coil content (Figure 2a). Similarly, GOR IV server analysis of the STAB2-rs2271637 variant shows a shift in the proportions of alpha helix, extended strand, and random coil secondary structures. Compared to the wild type (leucine), the mutant type (valine) exhibits a slight decrease in an alpha helix from 18.54% to 18.11% and a small increase in both extended strands from 20.50% to 20.89% and random coil from 60.96% to 61.00% (Figure 2b).

**Figure 3:**
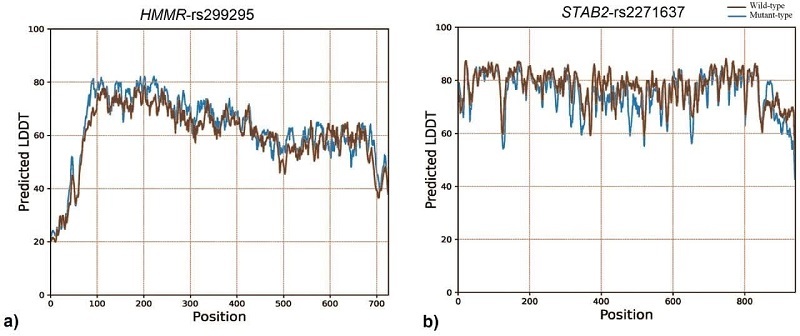
Rosetta LDDT prediction for HMMR-rs299295 and STAB2-rs2271637 variants: a) The predicted structure of the HMMR-rs299295 variant showed the mutant valine residue (blue) has a higher LDDT score than the wild-type alanine (red), indicating a better predicted local structural accuracy, b) The predicted structure of the STAB2-rs2271637 variant represented the wild-type leucine (red) has a higher LDDT score than the mutant valine (blue), suggesting a better predicted local structural accuracy for the wild-type protein.

The LDDT prediction is a valuable tool for assessing the reliability of protein structure models, particularly in terms of their local structural accuracy. The LDDT score measures how well the predicted structure of a protein matches the actual (experimental) structure. The LDDT score ranges from 0–100, where higher scores indicate better agreement between the predicted and actual structures. Constrained by the maximum acceptable length of amino acid sequences (1000 residues) for structural analysis using Rosetta's LDDT prediction server, only residues 1552–2552 of STAB2 were evaluated for LDDT score prediction. Rosetta's LDDT prediction for the *HMMR*-rs299295 variant suggests the mutant valine residue achieves a better score compared to the wild type (Figure 3a). In contrast, the *STAB2*-rs2271637 variant shows the opposite trend, with the wild-type leucine having a higher score than the mutant (Figure 3b).

Using the homology-based performance server, we analyzed the alanine into a valine mutation at position 485 in the *HMMR*-rs299295 variant and found that the mutant residue is larger than the wild-type. This mutation occurs within a region necessary for interaction with family with sequence similarity 83 member D (FAM83D). The change in amino acid properties could disrupt this region and impair HMMR function. The homology-based performance server predicted potential functional consequences for the L2401V mutation in *STAB2*-rs2271637. The introduced valine is sterically smaller than the wild-type leucine, potentially affecting protein conformation. This mutation resides within domain 7 fasciclin-like hyaluronan receptor homolog family (FAS1) as annotated by UniProt. Disruption of this domain by the mutation could abolish the STAB2 function.

**Figure 4:**
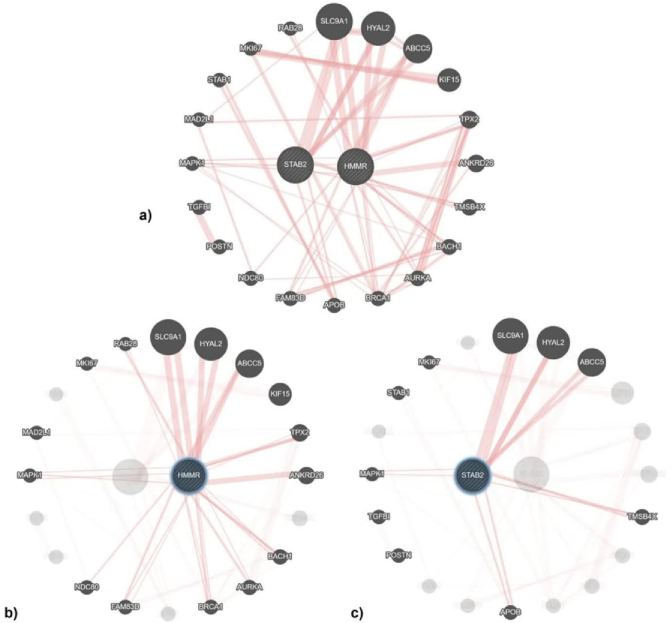
Protein physical interactions network of HMMR and STAB2: a) The network demonstrated that HMMR and STAB2 proteins, directly and indirectly, interact with nearly 20 other proteins, b) HMMR interacts both directly and physically interacts with 12 other proteins, c) STAB2 directly interacts with 6 proteins. HMMR: Hyaluronan mediated motility receptor, STAB2: Stabilin 2, TPX2: TPX2 microtubule nucleation factor, ANKRD26: Ankyrin repeat domain containing 26, TMSB4X: Thymosin beta 4 X-linked, BACH1: BTB domain and CNC homolog 1, AURKA: Aurora kinase A, BRCA1: BRCA1 DNA repair associated, APOB: Apolipoprotein B, FAM83D: Family with sequence similarity 83 member D, NDC80: NDC80 kinetochore complex component, POSTN: Periostin, TGFBI: Transforming growth factor beta-induced, MAPK1: Mitogen-activated protein kinase 1, MAD2L1: Mitotic arrest deficient 2 like 1, STAB1: Stabilin 1, MKI67: Marker of proliferation Ki-67, RAB28: RAB28, member RAS oncogene family, SLC9A1: Solute carrier family 9 member A1, HYAL2: Hyaluronidase 2, ABCC5: ATP binding cassette subfamily C member 5, KIF15: Kinesin family member 15.

The results from studying the physical interactions of HMMR and STAB2 indicated that these proteins have direct and indirect interactions with nearly 20 other proteins (Figure 4a). HMMR directly interacts with 12 proteins including member 28 RAS oncogene family (RAB28), solute carrier family 9 member A1 (SLC9A1), hyaluronidase 2 (HYAL2), ATP-binding cassette subfamily C member 5 (ABCC5), Kinesin family Member 15 (KIF15), TPX2 microtubule nucleation factor (TPX2), ankyrin repeat domain containing 26 (ANKRD26), BTB domain and CNC homolog 1 (BACH1), aurora kinase A (AURKA), breast cancer type 1 susceptibility protein (BRCA1), family with sequence similarity 83 member D (FAM83D), NDC80, and mitogen-activated protein kinase 1 (MAPK1) (Figure 4b). Following STAB2 directly interacts with 6 proteins SLC9A1, HYAL2, ABCC5, thymosin beta 4 X-linked (TMSB4X), apolipoprotein B (APOB), and MAPK1 (Figure 4c). Among these physical interactions, 4 proteins ABCC5, HYAL2, SLC9A1, and MAPK1 are common to both HMMR and STAB2.

**Table 2:** Genotype and allele frequencies of *HMMR*-rs299295 and *STAB2*-rs2271637

**Genotypes/alleles**		**Control** **(n = 98)**	**Case group I** **(BPH; n = 250)**	**OR** **(95% CI)**	**P-value**				**Case group II** **(cancer; n = 250)**		**OR** **(95% CI)**	**P-value**			
					**BLR**	**HWE**	**Chi^2^**	**FE**				**BLR**	**HWE**	**Chi^2^**	**FE**
***HMMR*-rs299295**															
	**CC**	78 (79.6)	50 (20)	Reference	-	0.234	0.0001	0.0001	96 (38.4)	Reference		-	0.282	0.0001	0.0001
	**CT**	15 (16)	138 (55.2)	0.073 (0.046-0.116)	< 0.001	--	--	--	86 (34.4)	0.224 (0.143-351)		< 0.001	--	--	--
	**TT**	5 (4.4)	62 (24.8)	22.433 (11.004-45.731)	< 0.001	--	--	--	68 (27.2)	12.814 (6.479-25.343)		< 0.001	--	--	--
	**CT+TT**	20 (20.4)	200 (80)	15.608 (10.068-24.153)	< 0.001	--	--	--	154 (61.6)	6.259 (4.189-9.333)		< 0.001	--	--	--
	**C-allele**	171 (87.6)	238 (47.6)	Reference	--	--	--	--	278 (55.6)	Reference		--	--	--	--
	**T-allele**	25 (12.4)	262 (52.4)	7.777 (6.655-10.695)	< 0.001	--	0.0001	0.0001	222 (44.4)	5.641 (4.100-7.762)		< 0.001	--	0.0001	0.0001
***STAB2*-rs2271637**															
	**CC**	57 (58.8)	68 (27.2)	Reference	**-**	0.196	0.0001	0.0001	110 (44)	Reference		**-**	0.164	0.0001	0.0015
	**CG**	39 (40)	175 (70)	3.783 (2.592-5.522)	< 0.001	--	--	--	125 (50)	1.670 (1.670-1.164)		0.005	--	--	--
	**GG**	2 (1.2)	7 (2.8)	2.246 (1.125-4.484)	0.022	--	--	--	15 (6)	2.585 (1374-4.863)		0.003	--	--	--
	**CG+GG**	41 (41.2)	182 (72.8)	1.954 (1.620-2.358)	< 0.001	--	--	--	140 (56)	1.348 (1.129-1.609)		< 0.001	--	--	--
	**C-allele**	153 (78.8)	311 (62.2)	Reference	--	--	--	--	345 (69)	Reference		--	--	--	--
	**G-allele**	43 (21.2)	189 (37.8)	2.259 (1.706-2.990)	< 0.001	--	0.0001	0.0001	155 (31)	1.670 (1.254-2.223)		< 0.001	--	0.0001	0.0001
Data presented as n (%). OR: Odds ratio, CI: Confidence interval, BLR: Binary logistic regression, HWE: Hardy-Weinberg equilibrium (it was evaluated using a χ² test), Chi2: Chi-square test (genotypes with df = 2 and for alleles with df = 1), FE: Fisher's exact test, HMMR: Hyaluronan-mediated motility receptor, STAB2: Stabilin-2, BPH: Benign prostatic hyperplasia															

## 4. Discussion

The *HMMR* and *STAB2* are genes encoding cell surface receptors that physically interact with ECM components, particularly HA. HMMR functions are multifaceted and structurally composed of a large extracellular domain, a single transmembrane domain, and a C-terminal cytoplasmic tail. The structure of HMMR includes several functional domains that facilitate its interaction with HA (7).

Functionally, HMMR is involved in various cellular processes, particularly those related to cell movement and division (10). It interacts with HA in the ECM to promote cell motility and proliferation, processes that are vital during tissue repair and remodeling. In cancer, HMMR overexpression has been associated with increased tumor aggressiveness and metastasis, as it activates several signaling pathways, including MAPK and PI3K/Akt, that drive cell growth and survival (7).

The STAB2 encodes a large multifunctional transmembrane protein that spans a region of approximately 248 kbp. The presence of a large extracellular domain, a single transmembrane domain, and a relatively short cytoplasmic tail is characteristic of this protein. It comprises 2551 amino acids, featuring multiple domains, including epidermal growth factor-like domains, Fasciclin domains, and X-linked 2 domain (X2) repeats. These domains are crucial for their function in ligand binding and endocytosis (19). Functionally, STAB2 plays a significant role in the clearance of glycosaminoglycans, such as HA. It acts as a scavenger receptor that binds and internalizes this molecule, facilitating its lysosomal degradation (12, 13).

In the context of cancer, *HMMR* and *STAB2* have been implicated in tumor progression and metastasis. Its role in the interaction with HA is particularly relevant, as this glycosaminoglycan is known to influence tumor cell proliferation, migration, and invasion (7, 11, 12). Dysregulation of these gene expressions has been observed in various cancers, including prostate cancers. Several variants of *HMMR* have been reported, and these polymorphisms have been linked to various cancer types (20). One of the *HMMR* gene's missense polymorphisms is *HMMR*-rs299295, which causes alanine to valine substitution at residue 485 (A485V). The results of the bioinformatics analysis revealed that this SNP induces a structural alteration in the protein (Figure 2a), and this structural change results in a protein configuration with higher energy levels compared to the wild-type. This increase in energy suggests that the modified protein structure is likely more stable than the original wild-type structure (Figure 3a). Such stability changes can have significant implications for the protein's function and interactions within the cell, potentially impacting various biological processes and contributing to disease pathogenesis. In addition, the investigations showed that this amino acid change is located in the 365–546 regions, which is an important part of the interaction with the family with sequence similarity 83 member D (FAM83D) protein. FAM83D is a key player in several cellular functions, such as cell cycle progression (21), protein localization to the mitotic spindle (22), and intracellular signaling (23).

HMMR, a protein essential for cell division and a known promoter of tumor development (8), has been implicated in targeting FAM83D to the mitotic spindle (10). Previous studies have shown that the inhibition of HMMR severely disrupts the assembly and integrity of the mitotic spindle (24). Our research revealed a significant link between the T mutant allele of the *HMMR*-rs299295 missense variant and increased risk in both groups compared to the control group (Table II). Based on the identified molecular and functional associations of HMMR and FAM83D with mitosis, we proposed that the *HMMR*-rs299295 missense variant encourages prostate neoplasm and progression by enhancing cell cycle progression via binding with HMMR and FAM83D.* STAB2*-rs2271637 is a missense variant of the *STAB2* gene, encoding a protein that functions as a receptor for HA.

Computational analysis has indicated that the *STAB2*-rs2271637 is predicted to be possibly damaging, with the capacity to alter the secondary structure of the protein (Figure 2b). STAB2 has 7 FAS1 domains that are involved in the binding of ECM components and different ligands including HA. The *STAB2*-rs2271637 (L2401V) missense variant is located within domain 7 of the FAS1 region (residues 2311–2446), which may alter the HA interaction's function of the STAB2 receptor.

The results of the gene network interaction analysis revealed that the *HMMR* and *STAB2* genes exhibit co-directly physical interactions with 4 distinct proteins, including ABCC5, HYAL2, SLC9A1, and MAPK1. MAPK1, also known as mitogen-activated protein kinase 1, is a serine/threonine kinase that plays a crucial role in various cellular functions, particularly in the MAPK/ERK pathway. This protein regulates processes such as cell proliferation, differentiation, survival, and invasion. In prostate cancer, MAPK1 promotes oncogenic pathways, contributing to cancer cell proliferation and metastasis (25). HYAL2 is an enzyme that plays a crucial role in the degradation of HA. While the direct link between HYAL2 and prostate cancer is not explicitly detailed, the evidence supports HYAL2 role in the progression of some cancers such as bladder and breast cancer (26).

This evidence can provide a convincing rationale for investigating HYAL2 and its contribution to prostate cancer. The ABCC5, also known as MRP5, is part of the ATP-binding cassette transporter superfamily and has been implicated in multiple drug resistance. ABCC5 emerges as a low critical factor in the progression of prostate cancer (27). The *SLC9A1* gene encodes the NHE1 protein, a sodium/hydrogen exchanger isoform 1, which plays a crucial role in regulating intracellular pH and volume in mammalian cells. Abnormalities in intracellular pH regulation have been linked to various pathological conditions, including cancer (28).

Although the provided references do not directly address the association of prostate neoplasm with the ABCC5, HYAL2, and SLC9A1 proteins, it appears that the *HMMR*-rs299295 and *STAB2*-rs2271637 variants may play a role in the growth, proliferation, and metastasis of prostate neoplasm. This effect is likely mediated through the disruption of the physical interaction between these 2 genes and the MAPK1 protein. According to the findings of this study and the effect of SNPs *HMMR*-rs299295 and *STAB2*-rs2271637 in the incidence and progression of prostate neoplasm, studies in a larger sample size and across more ethnicities are suggested to introduce these polymorphisms as a molecular marker.

## 5. Conclusion

Our study found a significant association between the mutant T allele in *HMMR*-rs299295 (A485V) and the mutant G allele in *STAB2*-rs2271637 (L2401V) variants and an increased risk of prostate neoplasm, including both groups (BPH, prostate cancer) compared to control men. Bioinformatic analysis showed these variants may disrupt protein structure and subsequently domains of the FAM83D and 7 FAS1 in HMMR and STAB2, respectively. These changed variants may potentially impact MAPK1 physical interaction, possibly contributing to prostate neoplasm progression.

##  Data Availability

The datasets generated and analyzed during the current study are available from the corresponding author upon reasonable request.

##  Author Contributions

A. Hosseinzadeh Colagar was involved in the conceptualization, methodology, and software sections. H. Abdulhadi Saleh Albdairi contributed to the material preparation, data curation and analysis, and preparation of the original draft. All authors approved the final manuscript and take responsibility for the integrity of the data.

##  Conflict of Interest 

The authors declare that there is no conflict of interest.

## References

[bib1] Bellos TC, Tzelves LI, Manolitsis IS, Katsimperis SN, Berdempes MV, Skolarikos A, et al (2022). Frailty and benign prostatic hyperplasia: The thrilling underlying impact. Arch Ital Urol Androl.

[bib2] Liss MA, Leach RJ, Sanda MG, Semmes OJ (2020). Prostate cancer biomarker development: National Cancer Institute's early detection research network prostate cancer collaborative group review. Cancer Epidemiol Biomarkers Prev.

[bib3] Sroka IC, Chopra H, Das L, Gard JM, Nagle RB, Cress AE (2016). Schwann cells increase prostate and pancreatic tumor cell invasion using laminin binding A6 integrin. J Cell Biochem.

[bib4] Hantash J (2018). The use of polysulfated polysaccharides heparin like compounds, glycosaminoglycans and vitamin B17 as a possible treatment for prostate cancer. Med Hypotheses.

[bib5] Samaržija I, Konjevoda P (2023). Extracellular matrix-and integrin adhesion complexes-related genes in the prognosis of prostate cancer patients’ progression-free survival. Biomedicines.

[bib6] Skarmoutsos I, Skarmoutsos A, Katafigiotis I, Tataki E, Giagini A, Adamakis I, et al (2018). Hyaluronic acid and hyaluronidase as possible novel urine biomarkers for the diagnosis of prostate cancer. Med Oncol.

[bib7] Carvalho AM, Reis RL, Pashkuleva I (2023). Hyaluronan receptors as mediators and modulators of the tumor microenvironment. Adv Healthc Mater.

[bib8] He Z, Mei L, Connell M, Maxwell CA (2020). Hyaluronan mediated motility receptor (HMMR) encodes an evolutionarily conserved homeostasis, mitosis, and meiosis regulator rather than a hyaluronan receptor. Cell.

[bib9] Jiang X, Tang L, Yuan Y, Wang J, Zhang D, Qian K, et al (2022). NcRNA-mediated high expression of HMMR as a prognostic biomarker correlated with cell proliferation and cell migration in lung adenocarcinoma. Front Oncol.

[bib10] Zhang X, Huang D, Li K, Han C, Li H, Li C, et al (2023). Hmmr acts as a key regulator in the ADSCs proliferation and mitosis. Stem Cell Rev Rep.

[bib11] Hare AK, Harris EN (2015). Tissue-specific splice variants of HARE/Stabilin-2 are expressed in bone marrow, lymph node, and spleen. Biochem Biophys Res Commun.

[bib12] Weigel PH (2020). Systemic glycosaminoglycan clearance by HARE/stabilin-2 activates intracellular signaling. Cells.

[bib13] Harris EN, Cabral F (2019). Ligand binding and signaling of HARE/Stabilin-2. Biomolecules.

[bib14] Park S-Y, Yun Y, Lim J-S, Kim M-J, Kim S-Y, Kim J-E, et al (2016). Stabilin-2 modulates the efficiency of myoblast fusion during myogenic differentiation and muscle regeneration. Nat Commun.

[bib15] Montazeri Dehbarez M, Kordi Tamandani D, Naeimi N, Vaziri Sh, Shekari M (2024). Association between GSK3β gene polymorphisms (rs334558 and rs3755557) with schizophrenia risk. J Genet Resour.

[bib16] Moudi E, Heydari M, Colagar AH (2023). CD44 rs13347C> T variants in 3'UTR and prostate neoplasms: A case-control study and bioinformatics approach. Int J Mol Cell Med.

[bib17] Green MR, Sambrook J (2012). Molecular cloning: A laboratory manual.

[bib18] Franz M, Rodriguez H, Lopes C, Zuberi K, Montojo J, Bader GD, et al

[bib19] Patten DA, Shetty S (2019). The role of stabilin-1 in lymphocyte trafficking and macrophage scavenging in the liver microenvironment. Biomolecules.

[bib20] Sukumar S, Krishnan A, Banerjee S, Singh V, Kumar A Advances in bioinformatics.

[bib21] Liu ZM, Yuan Y, Jin L (2024). FAM83D acts as an oncogene by regulating cell cycle progression via multiple pathways in synovial sarcoma: A potential novel downstream target oncogene of anlotinib. Discov Oncol.

[bib22] Li X, Sun C, Chen J, Ma JF, Pan YH (2022). Suppression of fam83d inhibits glioma proliferation, invasion and migration by regulating the akt/mtor signaling pathway. Transl Oncol.

[bib23] Wang D, Han S, Peng R, Wang X, Yang X-X, Yang R-J, et al (2015). FAM83D activates the MEK/ERK signaling pathway and promotes cell proliferation in hepatocellular carcinoma. Biochem Biophys Res Commun.

[bib24] Huang M, Ma X, Shi H, Hu L, Fan Z, Pang L, et al (2017). FAM83D, a microtubule-associated protein, promotes tumor growth and progression of human gastric cancer. Oncotarget.

[bib25] Hu B, Jin X, Wang J (2018). MicroRNA-212 targets mitogen-activated protein kinase 1 to inhibit proliferation and invasion of prostate cancer cells. Oncol Res.

[bib26] Dominguez-Gutierrez PR, Kwenda EP, Donelan W, O'Malley P, Crispen PL, Kusmartsev S (2021). Hyal2 expression in tumor-associated myeloid cells mediates cancer-related inflammation in bladder cancer. Cancer Res.

[bib27] Zhang H, Lian Z, Sun G, Liu R, Xu Y (2018). Loss of miR-516a-3p mediates upregulation of ABCC5 in prostate cancer and drives its progression. Onco Targets Ther.

[bib28] Guan X, Luo L, Begum G, Kohanbash G, Song Q, Rao A, et al (2018). Elevated Na/H exchanger 1 (SLC9A1) emerges as a marker for tumorigenesis and prognosis in gliomas. J Exp Clin Cancer Res.

